# Subjects develop tolerance to Pru p 3 but respiratory allergy to Pru p 9: A large study group from a peach exposed population

**DOI:** 10.1371/journal.pone.0255305

**Published:** 2021-08-19

**Authors:** Maria Luisa Somoza, Natalia Pérez-Sánchez, Laura Victorio-Puche, Laura Martín-Pedraza, Angel Esteban Rodríguez, Natalia Blanca-López, Eva Abel Fernández González, María Ruano-Zaragoza, Ana Prieto-Moreno Pfeifer, Enrique Fernández Caldas, Miriam Morán Morales, Francisco Javier Fernández Sánchez, José Damián López Sánchez, Teodorikez Wilfox Jiménez Rodríguez, José Luis Subiza Garrido-Lestache, Gabriela Canto Díez, Miguel Blanca Gómez, José Antonio Cornejo-García

**Affiliations:** 1 Allergy Department, Infanta Leonor University Hospital, Madrid, Spain; 2 Allergy Department, Hospital Regional Universitario de Málaga, Málaga-IBIMA, Málaga, Spain; 3 Allergy Department, Morales Meseguer General University Hospital, Murcia, Spain; 4 Allergy Department, Fundación para la Investigación e Innovación Biomédica (FIIB) de los Hospitales Universitarios Infanta Leonor y Sureste, Madrid, Spain; 5 Clinical Analysis Department, General University Hospital of Alicante, Alicante, Spain; 6 R&D Department, Inmunotek Laboratories, Madrid, Spain; 7 Allergy Department, General University Hospital of Alicante- ISABIAL, Alicante, Spain; 8 Allergy Department, Virgen de la Arrixaca Clinical University Hospital, El Palmar, Murcia, Spain; 9 Instituto de Investigación Biomédica de Málaga-IBIMA, Málaga, Spain; Srebrnjak Children’s Hospital, CROATIA

## Abstract

Peach tree allergens are present in fruit, pollen, branches, and leaves, and can induce systemic, respiratory, cutaneous, and gastrointestinal symptoms. We studied the capacity of peach fruit/Pru p 1, Pru p 3, Pru p 4, Pru p 7 and peach pollen/Pru p 9 for inducing symptoms following oral or respiratory exposure in a large group of subjects. We included 716 adults (aged 21 to 83 y.o.) exposed to peach tree pollen and fruit intake in the study population. Participants completed a questionnaire and were skin tested with a panel of inhalant and food allergens, including peach tree pollen, Pru p 9 and peach fruit skin extract. Immunoglobulin E antibodies (SIgE) to Pru p 1, Pru p 3, Pru p 4 and Pru p 7 were quantified. Sensitised subjects underwent oral food challenge with peach fruit and nasal provocation test with peach tree pollen and Pru p 9. The prevalence of sensitisation to peach fruit was 5% and most of these had SIgE to Pru p 3, with a very low proportion to Pru p 4 SIgE and no SIgE to Pru p 1 and Pru p 7. In only 1.8%, anaphylaxis was the clinical entity induced. Cases with positive skin tests to peach and SIgE to Pru p 3 presented a good tolerance after oral challenge with peach fruit. The prevalence of skin sensitisation to peach tree pollen was 22%, with almost half recognising Pru p 9. This induced respiratory symptoms in those evaluated by nasal provocation. In a large population group exposed to peach fruit and peach tree pollen, most individuals were tolerant, even in those with SIgE to Pru p 3. A positive response to Pru p 9 was associated with respiratory allergy.

## Introduction

Allergies are a worldwide problem affecting people of all ages. Sensitisation to allergens may occur by inhalation, ingestion, or contact [1]. Allergens present in plants, fruits and seeds are the most commonly involved in food allergy [2]. A large retrospective study in the USA reported a food allergy prevalence of 0.7% [3]. In a multicentre study in Spain, 7.4% of the patients screened did also receive this diagnosis [4]. Most epidemiological surveys are based on case series, drawn from patients attending Allergy centers, with an overestimation of the prevalence. This varies depending on the population, location, diet, pollen sensitisation, age, and gender amongst other common variables [1–3].

Two types of sensitisation to plant allergens have been reported: type A, consisting of primary sensitisation by the oral route with secondary respiratory response, and type B, sensitisation by the respiratory airways with secondary response to food allergens of vegetal origin [1, 5].

One family of widely distributed allergens are the lipid transfer proteins (LTP) [1, 2]. In most instances, LTP allergens, like Pru p 3, induce type A sensitisation by the oral route. They may have a response after inhalation of closely related LTPs present in pollens [1]. Pru p 3 sensitisation is frequent in the Mediterranean basin but is found across Europe [6, 7]. Type B sensitisation is induced via the inhalatory route. Furthermore, LTPs from pollens like Art v 3 and Pla a 3, sometimes producing a secondary response to Pru p 3 LTP [8]. Both models may coexist in the same patient [9, 10].

Recent evidence indicates that in areas of peach tree (PT) orchards, there is a high prevalence of sensitisation and allergy to PT pollen [11]. Although classified as *entomophilous* we could assume the behaviour of PT pollen to be *anemophilous*, generating sensitisation in exposed children and adults.

Allergy to PT pollen is generating interest in numerous countries. Spain is the third largest peach producer worldwide, after China and the USA, and the largest producer in Europe, with 70% of the fruit consumed fresh [12]. PT represents a good model because pollens, fruits, leaves and other tree parts have all been reported as sources of allergens and many individuals may be exposed [9, 10, 13].

Allergens identified so far in peach are Pru p 1 (PR-10 protein), Pru p 2 (thaumatin-like), Pru p 3 (ns-LTP), Pru p 4 (profilin), Pru p 5 (Hev b 5-like), Pru p 7 (gibberellin-regulated protein) involved in most instances in food allergy, and Pru p 9 involved in respiratory allergy [11, 13].

In this study, we evaluated a large rural population group exposed to PT pollen and peach ingestion. We aimed to study the prevalence and characterise the sensitisation and allergenic profile and compare the two allergy models. Two allergens were used as markers: Pru p 3 for oral sensitisation and Pru p 9 for respiratory sensitisation [13]. In addition, we quantified specific IgE (SIgE) to Pru p 1, Pru p 4 and Pru p 7.

## Methods

### Subjects’ inclusion

We consecutively recruited adults of both genders, aged 21 to 83 years from the Village of Blanca (Murcia, South-East Spain) who volunteer and agreed to participate. We used phone calls, community meetings, and public advertisements including local TV for providing information of the study. Subjects were recruited between the 9^th^ of May 2016 and the 2^nd^ of March 2017 and evaluated in the Blanca Primary Care Center.

For evaluating the relationship between age and sensitisation to the most prevalent pollens, gender and clinical entities were stratified in three groups of age: 1) 21–40 y.o., 2) 41–60 y.o., 3) 61–83 y.o.

Subjects of both genders with systemic diseases, autoimmunity, mental handicap, or other diseases were excluded from the study as well as pregnant women.

A written informed consent was signed by the participants prior to the inclusion.

The study was approved by our institutional Ethics Committee (Comisión de ética de la investigación del Hospital Universitario Infanta Leonor y Hospital Virgen de la Torre) and Comité de ética de la investigación, Hospital General Universitario Gregorio Marañón.

### Study variables

All variables and categories used are shown in S1 Appendix. Participants were interviewed for taking the clinical history, underwent skin prick tests (SPTs) and provided blood samples. In two subgroups, oral food challenges (OFC) and nasal provocation tests (NPT) were done to assess the clinical relevance of Pru p 3, Pru p 1, Pru p 4, Pru p 7 and Pru p 9, respectively.

### Clinical entities

We included rhinitis, asthma, and conjunctivitis to be respiratory allergies. The chosen criteria were based on a validated questionnaire adapted to our study [11, 14]. Since our population does not follow the north and central European model, we considered people with oral plus extraoral manifestations (excluding urticaria and anaphylaxis) to have oral allergy syndrome (OAS) rather than pollen allergy syndrome [1]. If participants reported no symptoms and had good tolerance to peach, they were classified as tolerant, independently of the skin test and SIgE values [14]. Other food-induced clinical entities like oesophagitis, gastroenteritis or isolated diarrhoea were not included because of the very low prevalence in this population.

### Skin prick testing and specific IgE to Pru 3

SPTs were performed with a panel of food and inhalant allergens prevalent in the region [9] (see S2 Appendix). These were provided by Inmunotek^®^ (Madrid, Spain). Native Pru p 9 was obtained as described [13]. The ImmunoCAP^®^ test (Thermo Fisher Scientific, Wahtham, Massachussets, USA) was used to detect SIgE to Pru p 1, Pru p 3, Pru p 4 and Pru p 7 with values higher than 0.35 kU_A_/L indicative of the presence of antibodies.

### Nasal provocation test

NPTs were performed as described [15] in a sub-sample of 20 participants positive to PT pollen and Pru p 9. This included people with and without occupational exposure.

### Oral food challenge

Participants with Pru p 3 SIgE but who reported tolerance to peach underwent a single-blind OFC to assess tolerance, as previously described [16, 17]. If confirmed, an open exposure test was carried out.

### Statistical analysis

Quantitative variables were described by means, medians, standard deviations, and confidence intervals. Qualitative variables, expressed as absolute frequencies and percentages, were analysed using the chi-squared statistic or Fisher’s exact test when frequencies numbered five or less. The tests were done using the statistical Package SPSS 21. We also performed multivariable logistic regression using the Stepwise method for two different outcomes: peach-induced food allergy or respiratory allergy. Both models included all the study variables in the analysis. Data was analysed using the Logistic regression Stepwise method at a significance level of alfa = 0.05. Software used was SAS© v. 9.4.

## Results

### Study group characteristics

A total of 716 adults were included in the study. In our study group, no statistical differences in gender distribution were identified according to the three 20-year age brackets (21–40 y.o., 41–60 y.o., 61–83 y.o.) (S1 Fig). Most participants (80%) were involved in agricultural activities related to peach cultivation; other occupations included social services, manufacturing, administration, health care, education, and others. A detail of activities is shown in S1 Table.

The 87% of the participants had always lived in the village.

### Foods involved

**Fig 1:**
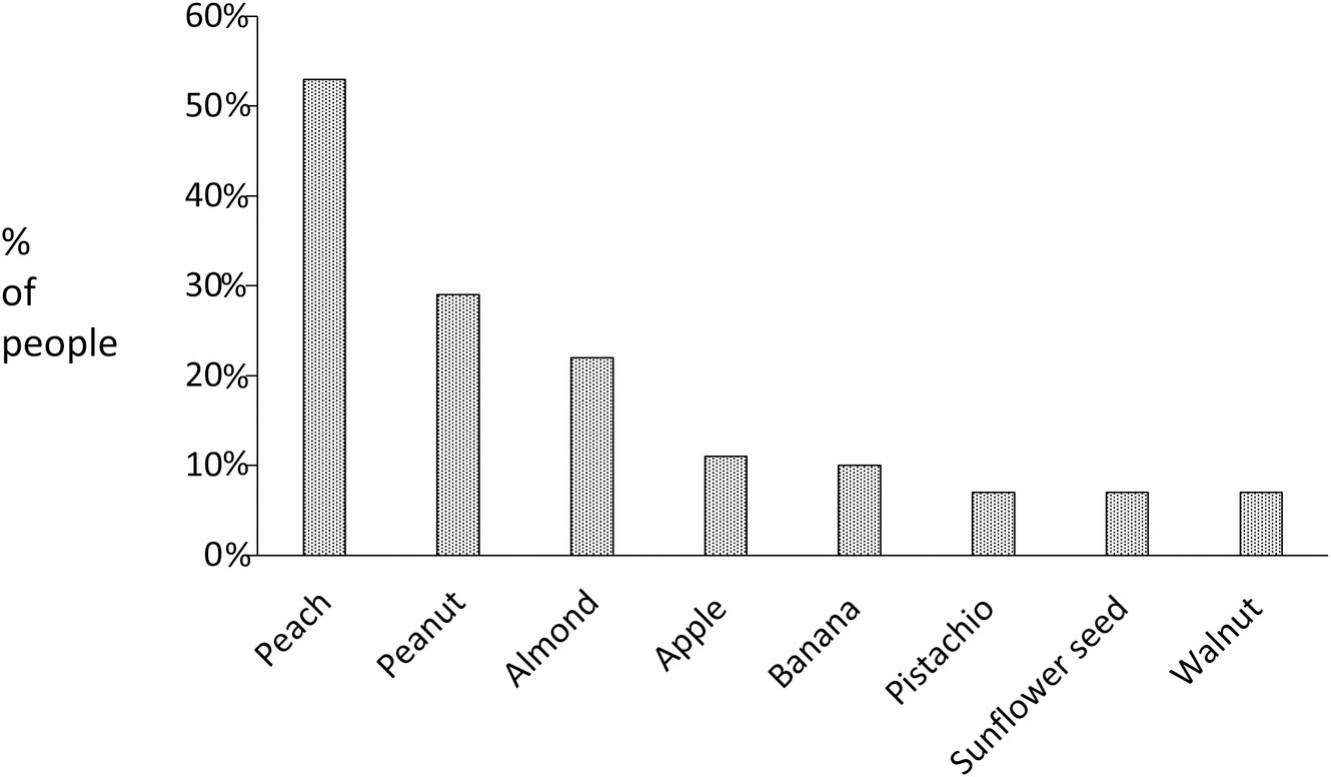
Fruit and nut intake in the study population, according to their preferences (% of participants reporting regular intake).

In terms of diet, the most frequent fruit or nut that participants reported regularly consuming was peach (53%), followed by peanut (30%) and almond (21%) (Fig 1). Virtually all participants (95%) had eaten peach since their early age, and 25% had eaten the fruit with the skin. Annual intake was estimated at 1–5 kg in 74% of the population, while 25% reported eating more than 5 kg per year.

### Skin prick test

**Fig 2:**
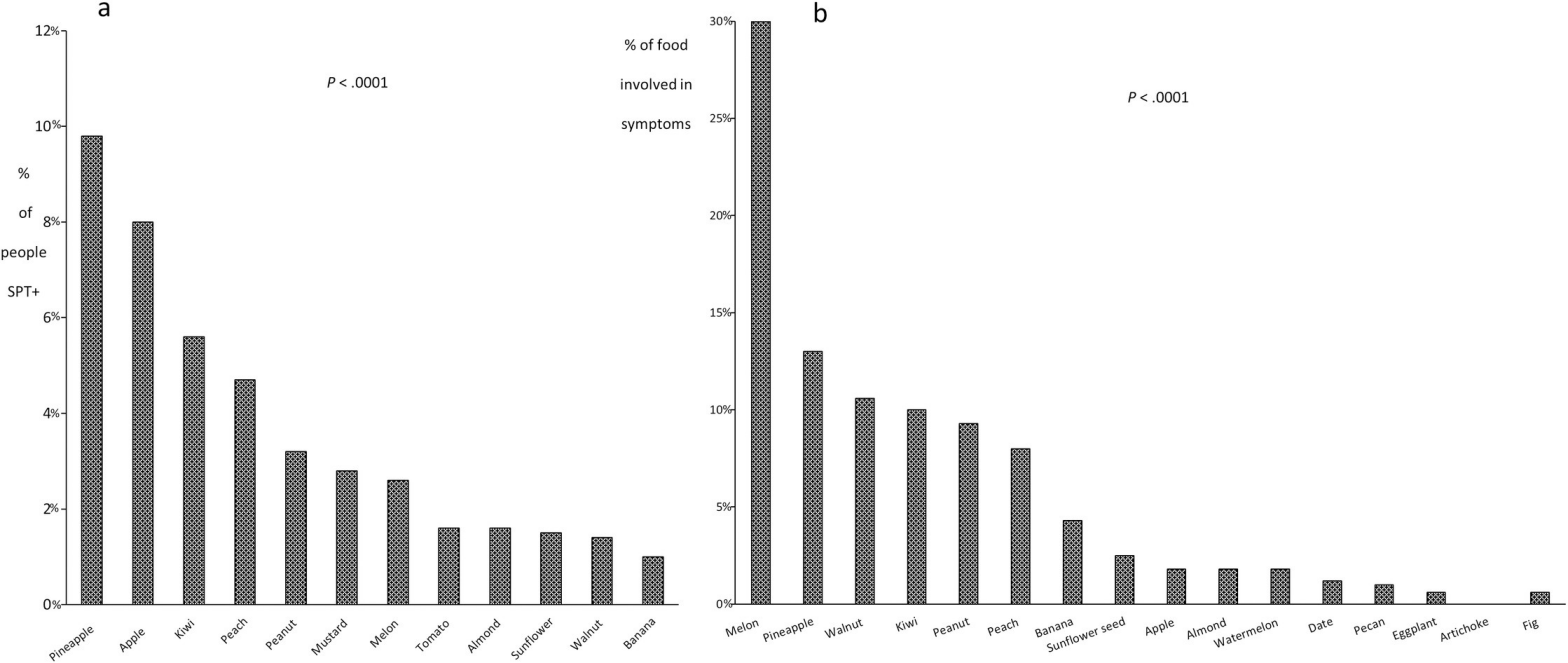
a) Percentage of subjects with positive skin prick tests to food allergens. b) Most commonly implicated fruits according to clinical history. SPT: skin prick test.

SPT was positive to at least one fruit allergen in 18% of the participants (Fig 2A). Pineapple was the most frequent (9.8%), while peach fruit positivity was found in less than 5%. There was no correlation between the food that induced symptoms and SPT. Melon was responsible for 30% of the cases with symptoms, whereas peach was involved in 8% (Fig 2B). Sensitisation decreased with age, but due the low number of positive SPT, statistical comparisons were not made.

### Food-induced clinical entities and SIgE to Pru p 3

Food-induced symptoms were reported in 17.8% of the participants, and OAS was diagnosed in 13.9%. Pollen sensitisation was significantly associated with OAS (*P* < .001), especially *Olea europaea* and PT pollens (*P* < .001), followed by *Salsola kali* (*P* < .001), *Phleum pratense* (*P* < .005), *Artemisia vulgaris* (*P* < .01), *Platanus acerifolia* (*P* < .05) and *Cupressus arizonica* (*P* < .05).

OAS to different fruits but good tolerance to peach was reported in 88 cases; 10 had extraoral symptoms in the gut, and 3 had an itchy nose or sneezing. The culprit foods are listed in S2 Table. Eleven cases (1.5% of participants) developed symptoms to peach as well as to other fruits (Table 1). Extraoral symptoms occurred in 2% of participants and consisted mostly of retrosternal or abdominal discomfort. SPTs showed positivity to peach fruit and Pru p 3 SIgE in two cases.

**Table 1:** Clinical characteristics of patients with OAS due to peach.

Case	Age	Gender	Clinical characteristics	Trigger foods	SPT Peach	IgE Pru p 1 kU_A_/L	IgE Pru p 3 kU_A_/L	IgE Pru p4 kU_A_/L	IgE Pru p 7 kU_A_/L
1	39	M	Tongue angioedema, oral pruritus	Peach, walnut	-	0.05	0	0.03	0.1
2	48	F	Oral pruritus, tongue angioedema	Tomato, melon, peach and apricot	+	0.03	**1.26**	**0.40**	0.1
3	32	M	Pharyngeal pruritus	Peach	-	0.02	0	0.01	0.1
4	49	M	Pharyngeal pruritus, difficulty swallowing	Peach, apricot	-	0.02	0	0.05	0.05
5	24	M	Lip angioedema, tongue pruritus	Melon, peach	-	0.02	0	0.05	0.01
6	33	M	Pharyngeal pruritus	Peach	-	0.1	0	0.02	0.03
7	21	F	Lip angioedema, tongue pruritus	Pineapple, melon, peach	+	0.1	**0.93**	0.02	0.01
8	33	M	Oral pruritus	Peach	-	0.05	0.1	**0.36**	0.03
9	22	M	Pharyngeal pruritus	Peanut, walnut, peach, banana, kiwi	-	0.2	0.05	0.02	0.01
10	36	F	Tongue pruritus	Peach	-	0.01	0	0.05	0.06
11	62	F	Oral pruritus, lip angioedema	Peach	-	0.05	0.05	0.02	0.02

M: male, F: female, SPT: skin prick test.

+: positive, -: negative.

Thirteen cases reported urticaria; peach was involved in 10 cases (1.4% of participants). The main characteristics are summarised in Table 2. Three participants had a positive SPT to peach fruit. SIgE to Pru p 3 was likewise detected in three cases, one of which was SPT-negative. Nine people showed contact urticaria (Table 2), including two who were tolerant to eating peach without skin.

**Table 2:** Clinical characteristics of participants with urticaria due to peach.

Case	Age	Gender	Trigger of urticaria; clinical characteristics	SPT peach	IgE Pru p 1 kU_A_/L	IgE Pru p 3 kU_A_/L	IgE Pru p 4 kU_A_/L	IgE Pru p 7 kU_A_/L
1	43	M	Physical contact with peach; good tolerance after eating without skin and to apple, peanut and walnut	+	0	**0.39**	0.03	0.1
2	52	F	Physical contact and after eating peach	-	0.00	0.07	0.1	0.1
3	43	M	Physical contact with peach; good tolerance after eating without skin	-	0.00	0.01	0.01	0.1
4	47	F	Physical contact with peach; good tolerance to apple	-	0.00	0.03	0.05	0.05
5	47	F	Physical contact with peach	-	0.00	0	0.05	0.01
6	22	F	Physical contact with peach and after eating peach and nectarine	+	0.00	0.01	0.02	0.03
7	38	M	Physical contact with peach	-	0.00	0.1	0.02	0.01
8	22	F	Physical contact with peach	+	0.04	**10.5**	**0.36**	0.03
9	29	M	Physical contact and after eating peach	-	0	**0.84**	0.02	0.01
10	34	M	After eating peach	-	0.02	0.2	0.05	0.06

M: male, F: female, SPT: skin prick test.

+: positive, -: negative.

Anaphylaxis appeared in 13 cases (1.8% of the participants); peach was involved in all of them (Table 3). In all but one case, participants were SPT-positive to peach and showed Pru p 3 SIgE.

**Table 3:** Cases with anaphylaxis induced by peach.

Case	Age	Gender	Symptoms and trigger	SPT peach	Pru p 1 IgE kU_A_/L	Pru p 3 IgE kU_A_/L	Pru p 4 IgE kU_A_/L	Pru p 7 IgE kU_A_/L
1	26	F	Nausea, vomiting and systemic pruritus after eating peach with peel or pomegranate	+	0.05	**4.86**	0.03	0.1
2	39	F	Facial angioedema, dyspnoea and systemic pruritus after eating peach and apple. Rhinitis and difficulty breathing around peach tree orchards	+	0.1	**2.55**	0.1	0.1
3	34	F	Generalised angioedema, systemic pruritus, and difficulty breathing after eating walnut. Gastrointestinal pain, systemic pruritus and angioedema of eyes after eating peach, nectarine or apricot	+	0.05	**3.08**	0.01	0.1
4	48	F	Systemic pruritus, nausea, vomiting, dizziness and hypotension after eating banana or drinking peach juice	+	0.05	0.35	0.05	0.05
5	62	F	Dyspnoea, facial angioedema and systemic pruritus after eating peach, sunflower seed or strawberry	-	0.05	0.12	0.05	0.01
6	25	F	Dyspnoea, generalised urticaria and difficulty breathing after eating peach, peanut, almond, walnut or sunflower seed	+	0.01	**13.7**	0.02	0.03
7	44	F	Systemic pruritus with facial angioedema and dyspnoea after drinking apricot juice and eating peanut or peach	+	0.05	**0.36**	0.02	0.01
8	33	F	Dyspnoea, generalised angioedema and hypotension after eating banana, peach, peanut or walnut	+	0.01	**7.75**	**0.36**	0.03
9	66	F	Generalised skin pruritus and difficulty breathing after eating peanut, peach or apple	+	0.05	**2.37**	0.02	0.01
10	23	F	Hypotension, generalised urticaria and systemic pruritus after eating walnut or peach	+	0.02	**17.8**	0.05	0.06
11	37	M	Difficulty swallowing, dysphonia and systemic pruritus after eating peach, peanut, sunflower seed, almond or pistachio.	+	0.03	**0.37**	0.02	0.02
12	47	F	Nausea, vomiting and systemic pruritus after drinking peach juice. Lip angioedema after eating walnut	+	0.03	**1.4**	0.02	0.03
13	35	F	Generalised pruritus, facial angioedema and difficulty breathing after eating peanut and peach	+	0.10	**29.3**	**0.36**	0.01

F: female, M: male, SPT: skin prick test.

### Specific IgE to Pru p 1, Pru p 4 and Pru p 7

When analysing the different clinical entities SIgE antibodies to Pru p 1 and Pru p 7 were not found. SIgE to Pru p 4 were found in five cases, 2 corresponding to OAS, 1 urticaria and 2 anaphylaxis. (see Tables 1–3).

### Tolerance to peach fruit and SIgE antibodies to Pru p 3

In most cases (n = 681, 95%), participants had negative SPTs to peach fruit, no detectable SIgE to Pru p 3, and good tolerance.

Analysing all cases with SIgE to Pru p 3 we found 39 cases, 22 were tolerant according to clinical history and 17 had OAS, urticaria or anaphylaxis. There were no significant differences in age, SIgE values to Pru p 3 or gender between tolerant and those with symptoms. Comparing within the SIgE group tolerant versus symptomatic, 91% tolerant had skin test positive to peach tree pollen versus the 65% of symptomatic (*P* < .04); for Pru p 9, 41% of tolerant were skin test positive versus the 6% of symptomatic (*P* < .01). There were no significant differences for the rest of pollens tested (S3 Table).

When same comparison was made in this group with the SPT to vegetal foods no statistical differences were seen in both groups (S3 Table).

### Oral food challenge with peach

From the 22 cases who had positive SIgE to peach but reported tolerance, twelve agreed to be challenged as described in the method section. These included cases of both genders with SIgE to Pru p 3 including high values (S4 Table).

In all instances, good tolerance was observed confirming the data reported in the clinical history. Matching these cases with those no challenged, they were similar in age, gender, skin testing to pollen or vegetal allergens.

The multivariable logistic regression showed an association between the outcome of peach allergy with being a woman, sensitisation to apple, positive SPT to any fruit, and antibodies to Pru p 3. S5 Table shows the odds ratios and confidence intervals.

### Pollen sensitisation

**Fig 3:**
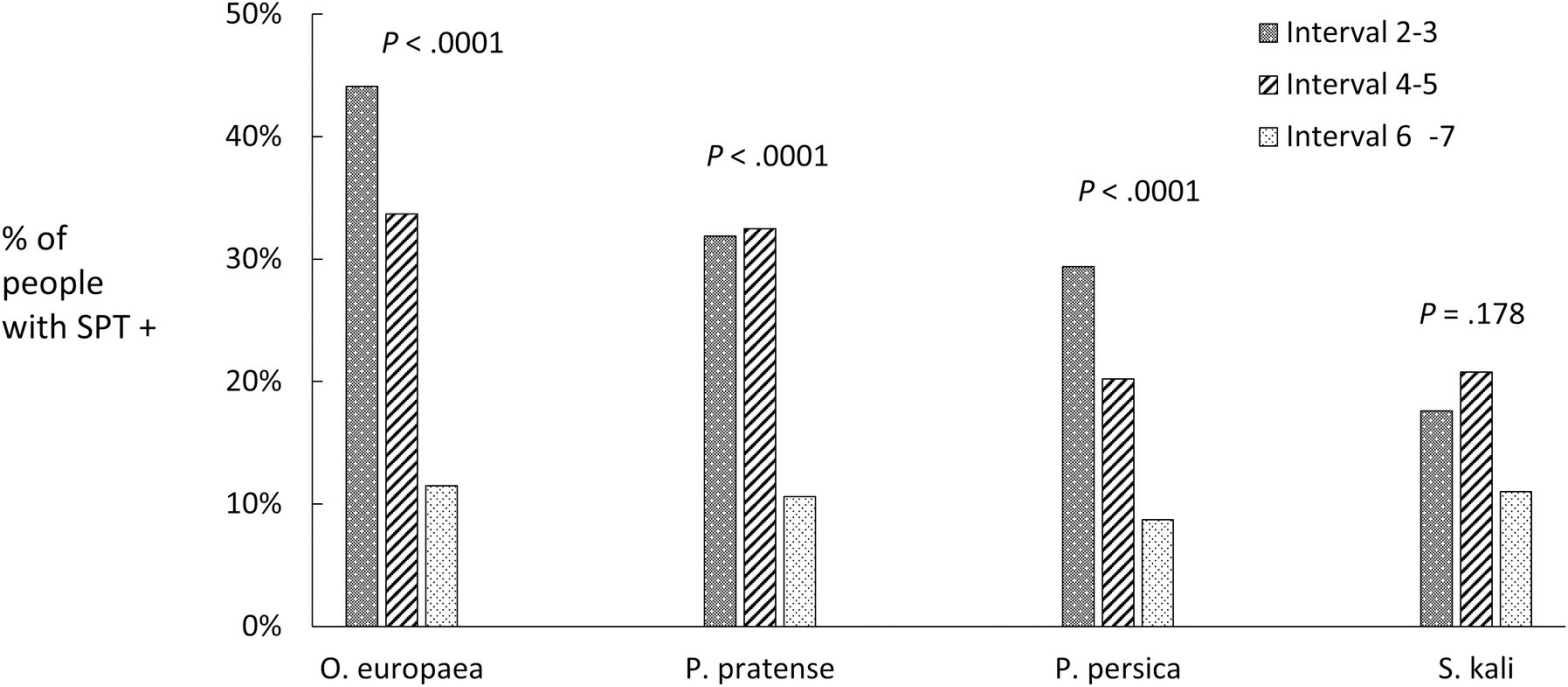
Sensitisation to the four most prevalent pollen allergens, by age groups. SPT: skin prick test, interval 2–3: 21–40 y.o., interval 4–5: 41–60 y.o., interval 6–7: 61–83 y.o.

Half the total sample was sensitised to at least one pollen; 22% had positive SPT to PT pollen (S2 Fig). There were significant differences in sensitisation to the pollens tested (*P* < .0001). Sensitisation to *A*. *vulgaris* was 10%, and to *P*. *acerifolia*, 7.4%. Sensitisation occurred in 57.1% of the women compared to 49% of men (*P* < .01). Prevalence of sensitisation trended downward with age for the three most prevalent pollens tested although not significant for *S*. *kali* (Fig 3). The 89% were sensitised to more than one pollen (S3 Fig), while 8.9% were sensitised to a single pollen (S4 Fig). Sensitisation to other allergens was 7.2% for *Dermatophagoides pteronyssinus*, 6% for cat epithelia, 5.4% for dog epithelia and 3.4% for *Alternaria alternata*.

Sensitisation to Pru p 9 occurred in the 38% of the cohort, with no statistical differences among age groups.

### Clinical entities related to inhalants

**Fig 4:**
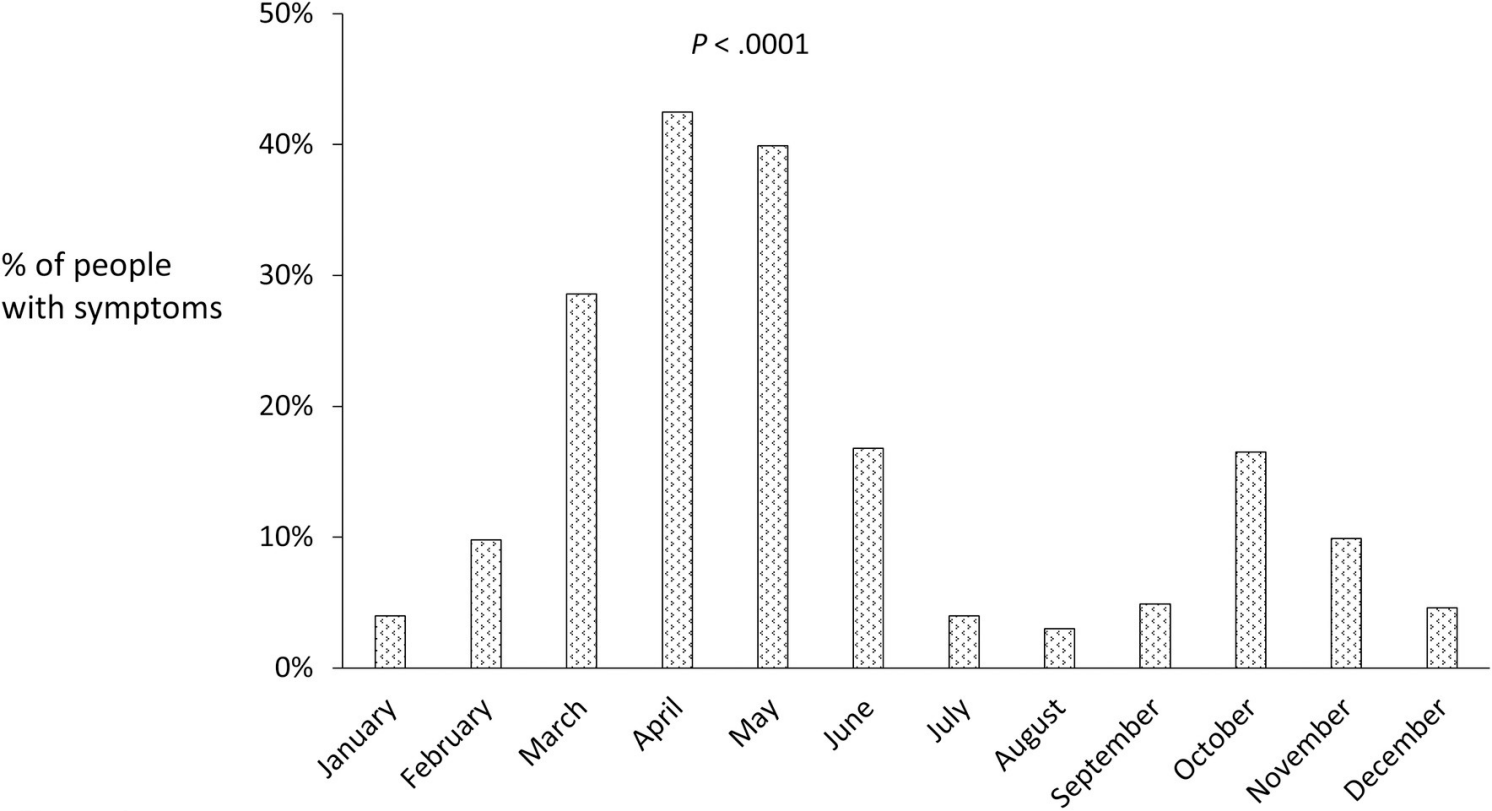
Percentage of participants presenting symptoms of respiratory allergy, by months of the year.

Rhinitis occurred in 62.6% of the cases, conjunctivitis in 52.9% and asthma in 21%. Most cases with asthma were associated with rhinitis or rhinoconjunctivitis. Asthma without rhinitis occurred in 11 cases and conjunctivitis alone in 10 cases. Combined respiratory symptoms appeared in 62% of the participants. Participants reported symptoms most frequently in spring, especially April, with more than 40% of the population affected. A smaller peak (less than 20% of the population) was apparent in October and November (Fig 4). The month(s) when symptoms appeared were related to the pollen type: March was associated with sensitisation to PT pollen; April and May, to *P*. *pratense*; and May, to *O*. *europaea*. Although *S*. *kali* induced more symptoms in autumn the difference was not significant. The prevalence in different age groups showed a downward gradient, which was highly significant for rhinitis (*P* < .001), conjunctivitis (*P* < .001) and for asthma (*P* < .01) (S5 Fig).

### Peach tree pollen and Pru p 9 in respiratory symptoms

We selected another subgroup of 20 cases (12 women, 8 men) sensitised to both PT pollen and Pru p 9 for NPTs. Results are shown in S6 Table. Half had occupational exposure, while the other half were exposed only occasionally. Results showed that 18 of the 20 were positive to PT pollen and 18 to Pru p 9. The two cases that were NPT-negative to PT pollen were positive to Pru p 9 challenge.

Multivariable logistic regression showed several variables associated with respiratory allergies: being a woman, sensitisation to pollen and those in the youngest group (21–40 and 41–60) compared to the oldest (S5 Table).

## Discussion

Our study includes a large number of adults, aged 21 to 83 years, from a rural population with high exposure to peach fruit and pollen. This was the village of Blanca (Murcia Region, Spain). The 80% were involved in PT agriculture, and more than 90% had eaten peach and other fruits [18].

Our group has previously reported sensitisation to PT pollen and resulting symptoms in people with and without occupational exposure, including children and adolescents [11, 13]. As the PT is mainly *entomophilous*, it is reasonable to assume that our population, in close contact with PT orchards, was exposed to PT pollen or small particles carrying pollen fragments. Unpublished data from our group showed 6–8 grains/m^3^ of PT pollen on the roofs of the village in late February and March, the period of PT pollen release.

In our study, the prevalence of sensitisation to peach fruit and other food allergens based on SPT to fruits was 17.8%. These figures are higher than those found in other studies based in the general population [19–21].

Regarding the clinical entities, we considered OAS instead of pollen food syndrome because our population, a rural community located in southern Spain, differs from those in central European countries with regard to pollen exposure and fruit intake. The other clinical entities were urticaria and anaphylaxis. OAS was reported in 13.9% of our population, while associated extraoral symptoms were present in just a few, mainly the gut and in two cases rhinitis, as reported elsewhere [22].

The proportion of people who were SPT-positive to pollens and developed OAS was 18%. These numbers are lower than those reported in the literature. In the Northern hemisphere, up to 70% of people who are allergic to birch pollen also develop an allergy to apple and other *Rosaceae* fruits [1], and in Korea, 41% of cases with pollinosis suffered from food allergy, with a plurality of cases considered OAS [23].

In our studies, the involvement of LTPs in food allergy was low, as published [24], even though our data were obtained from a population that we considered of high exposure. Peach was exclusively involved in just 11 cases with OAS, although this was by far the most commonly eaten fruit in the population. The contribution of Pru p 3 and Pru p 4 specific IgE was very low and Pru p 1 and Pru p 7 were negative in all cases.

Contact urticaria occurred in 28% of the sample, a much lower proportion than that reported by Cuesta-Herranz *et al*, also in Spain [25] and similar to other studies [26]. We did not include cases that developed pruritus in the absence of any other skin symptoms because this is a very common manifestation, and in our experience, it is not related to peach sensitisation. The contribution of Pru p 3, and Pru p 4 was also low with Pru p 1 and Pru p 7 being negative in all cases. There was no significant association between sensitisation to pollens (including PT pollen) and contact urticaria.

Although infrequent, anaphylaxis was the most commonly reported clinical entity, in all but one of these participants showing Pru p 3 SIgE. Only in two cases Pru p 4 SIgE were found with no detectable specific antibodies to Pru p 1 and Pru p 7. Although a significant association between pollen sensitisation and fruit-induced anaphylaxis has been reported [27], our data did not support this previous finding.

In our cohort, the vast majority (95.5%) were SPT-negative to peach and showed negligible SIgE to Pru p 3. In only 40 cases SIgE to Pru p 3 were found, and more than the half had good tolerance, verified by controlled exposure, although in a limited number of cases. These data, in a highly exposed population indicate that there is oral tolerance that extended not only to all negative skin tests but also to cases with specific IgE antibodies to Pru p 3. As this fruit is consumed since an early age in our cases, including pregnant and breastfeeding women, this may contribute to tolerance in subjects who had never developed symptoms throughout their lives. This has been shown with other food allergens including milk and peanut [28, 29].

Regarding tolerance in those with SIgE to Pru p 3, several studies support our findings. This has been reported for wheat, peanut, and other allergens [30, 31]. In a previous study carried out by our group, 6.9% of the adult population had SIgE to Pru p 3 but good tolerance [32]. Factors contributing to sensitisation without progressing to clinical disease are not well known [33].

Logistic regression analysis showed that being a woman, SPT-positive to vegetal foods and to apple were moderate risk factors for being allergic to peach fruit. However, SIgE antibodies to Pru p 3 yielded an odds ratio of 12.0 for this outcome.

Regarding pollens, one study in Spain showed that 40% to 63% of people in different regions are sensitised. Our result of 49%, in a sample drawn from the general population, is much higher than those reported in the Spanish allergy centers [34]. Our data are similar to the NHANES III study [35], although we carried out the research in a rural population, where prevalence is expected to differ.

In contrast to the low prevalence of food allergy and Pru p 3 sensitisation, the 22% was sensitised to PT pollen (one out of 5 cases), following olive tree and grass pollen as the most frequent by SPT. These data are consistent with previous studies performed by our group [11, 13].

In terms of the time of year, the months of March, April and May were the most important for respiratory symptoms, and late February and March specifically for PT pollen. Additionally, sensitisation significantly decreased with age, which is consistent with the data reported in the literature [36].

Our results corroborate the significant association between positive SPTs to pollens and the presence of rhinitis, asthma, and clinical symptoms [34, 37]. Sensitisation to pollens from early childhood is a risk factor for the development of rhinitis and asthma [37–39]. Previous research by our group in the same community but in children and adolescents showed high levels of sensitisation in this population [11].

In order to prove the relevance of Pru p 9 in inducing symptoms, we randomly selected a group of 20 participants sensitised to PT pollen, including those with and without occupational exposure. Results indicate that Pru p 9 is involved in the induction of respiratory symptoms, independently of exposure. The relevance of Pru p 9 in occupational respiratory allergy has been previously shown by our group [40]. The possibility that other PT pollen allergens are involved exists since in previous studies we have found subjects negative to Pru p 9 but with IgE to several allergens of different molecular weights. However, because we did not find Pru p 3 in pollen [13], it seems unlikely that this LTP could be involved in respiratory symptoms.

When we considered tolerance of peach intake as a dependent variable, we found that intolerance was associated with female gender, positive SPT to apple, sensitisation to any food, and SIgE antibodies to Pru p 3. Variables associated with respiratory allergy were age of 21 to 40 years and 41 to 60 years, female gender, and sensitisation to other pollens.

This study has several limitations: all cases with positive skin tests to Pru p 3 and a history of anaphylaxis were classified as such, even though it is possible that some may have developed tolerance over time. However, because they still tested positive on SPT and had relevant levels of Pru p 3 antibodies, we believe it is reasonable to consider them as responders. In addition, although the sensitivity for fruit allergens is not optimal, peach peel extract is highly allergenic in people with IgE antibodies to Pru p 3 [25, 40]. Another limitation is the evaluation of the skin test response in older people, whose skin response is known for its low reactivity [41].

Although we included a large group of subjects consecutively recruited from a population with high exposure to peach fruit and peach pollen, this sample was not representative of the total population since the percentage of the youngest and males in our study was slightly lower due to the difficulty to reach these subjects.

In summary, we provide new data on the prevalence of sensitisation and allergy to peach fruit, Pru p 1, Pru p 3, Pru p 4 and Pru p 7 as well as PT pollen and Pru p 9 in a large adult population with high levels of environmental exposure. Our results show a low proportion of people with sensitisation and allergy to peach and Pru p 3 but a remarkably high proportion for PT pollen and Pru p 9. Early life exposure may induce oral tolerance but does not seem to prevent inhalant allergy. The role of Pru p 1, Pru p 4 and Pru p 7 seems of little relevance.

## Supporting information

S1 Fig Participants by gender and age group: 2–3 (21–40 years old), 4–5 (41–60 years old), 6–7 (61–83 years old). (TIF)

S2 Fig Results of positive skin prick testing to the different pollens studied. SPT: skin prick test.
(TIF)

S3 Fig Number of pollens involved in cases with polysensitisation. (TIF)

S4 Fig Monosensitisation to different pollens. (TIF)

S5 Fig Clinical manifestations by age intervals. Interval 2–3: 21–40 y.o., interval 4–5: 41–60 y.o., interval 6–7: 61–83 y.o.
(TIF)

S1 Table Demographic characteristics of the study group. (DOCX)

S2 Table Clinical characteristics of cases with OAS. M: male, F: female.
(DOCX)

S3 Table Characteristics and sensitisation to pollen and food of subjects with SIgE to Pru p 3. (DOCX)

S4 Table Characteristics of cases tolerant to peach fruit with positive SIgE to Pru p 3. F: female, M: male, OAS: oral allergy syndrome.
(DOCX)

S5 Table Multivariable logistic regression analysis for peach-induced food allergy or respiratory allergy. CI: confidence interval; OR: odds ratio; SPT: skin prick test.
(DOCX)

S6 Table Characteristics of cases that underwent nasal provocation test to peach tree pollen and Pru p 9. R: rhinitis, RC: rhinoconjuctivitis, A: Asthma, Pollens for skin prick testing: O: Olea europaea, C: Cupresus arizonica, Pj: Parietaria judaica, Pp: Phleum pratense, S: Salsola kali, Pa: Platanus acerifolia, Av: Artemisia vulgaris. PTP: peach tree pollen, SPT: skin prick test, NPT: nasal provocation test. +: positive. -: negative. Positive NPT: >20% nasal volume fall and compatible symptoms.
(DOCX)

S1 Appendix Quantitative and qualitative variables. (DOCX)

S2 Appendix Allergens tested. (DOCX)

## Abbreviations

SIgE: specific immunoglobulin E
LTP: lipid transfer protein
PT: peach tree
y.o.: years old
SPT: skin prick test
OFC: oral food challenge
NPT: nasal provocation test
OAS: oral allergy syndrome
