# Supplementary figures and images for: Subjects develop tolerance to Pru p 3 but respiratory allergy to Pru p 9: A large study group from a peach exposed population
Source: PLoS One. 2021 Aug 19;16(8):e0255305. doi: 10.1371/journal.pone.0255305 (PMC8376049; doi:10.1371/journal.pone.0255305)

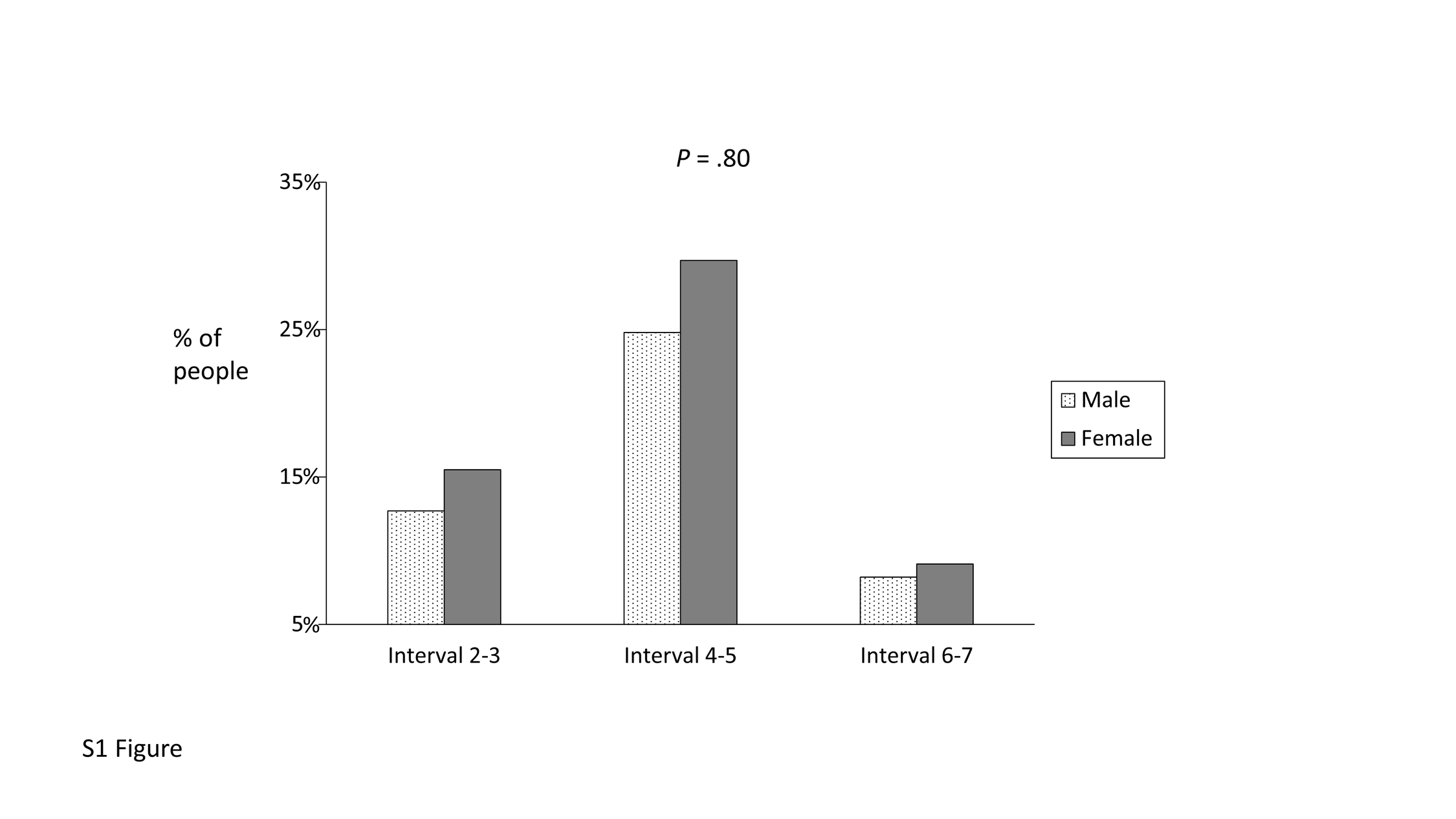

Supplement: S1 Fig — (TIF) [file pone.0255305.s001.tif]

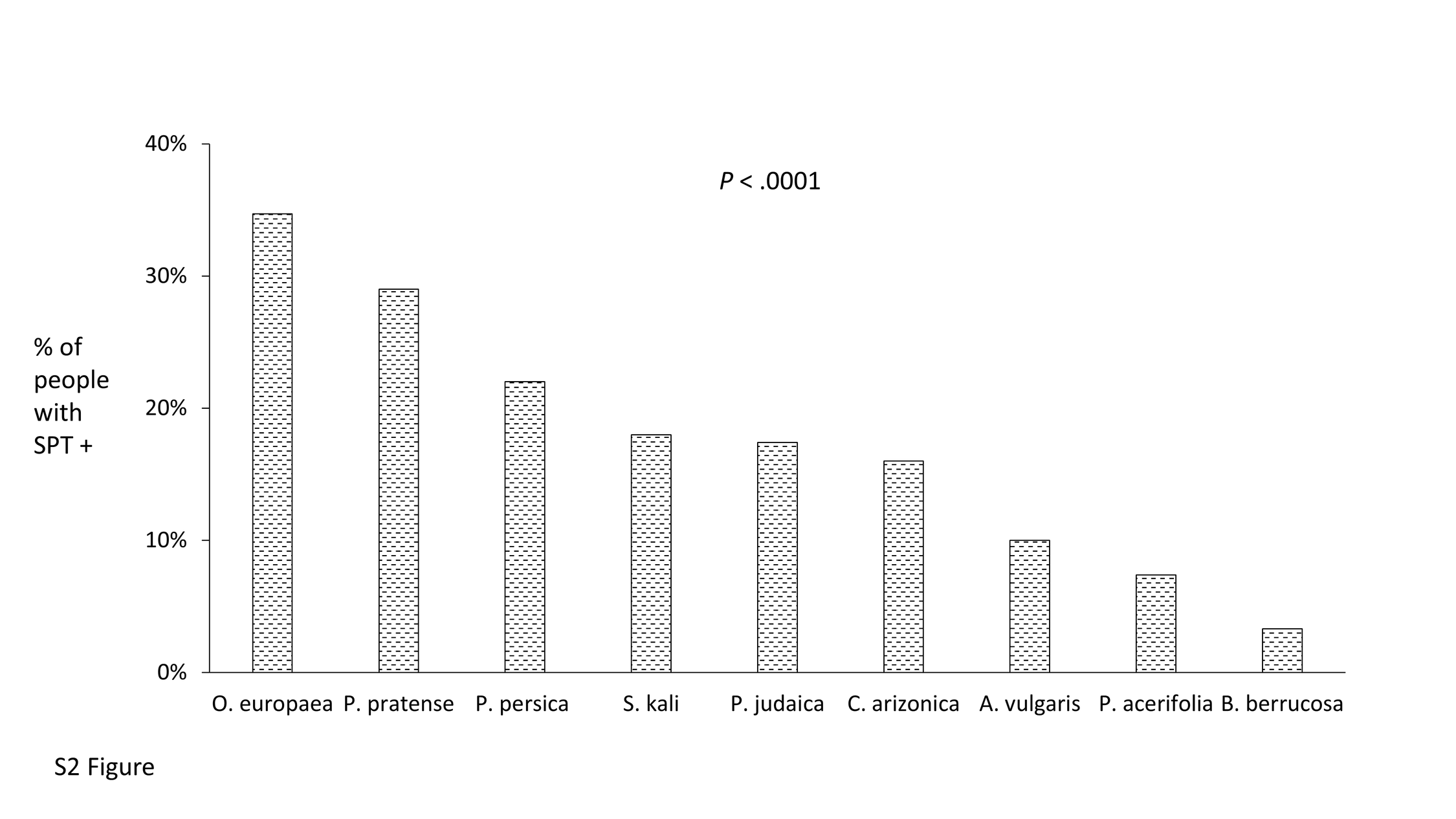

Supplement: S2 Fig — SPT: skin prick test. (TIF) [file pone.0255305.s002.tif]

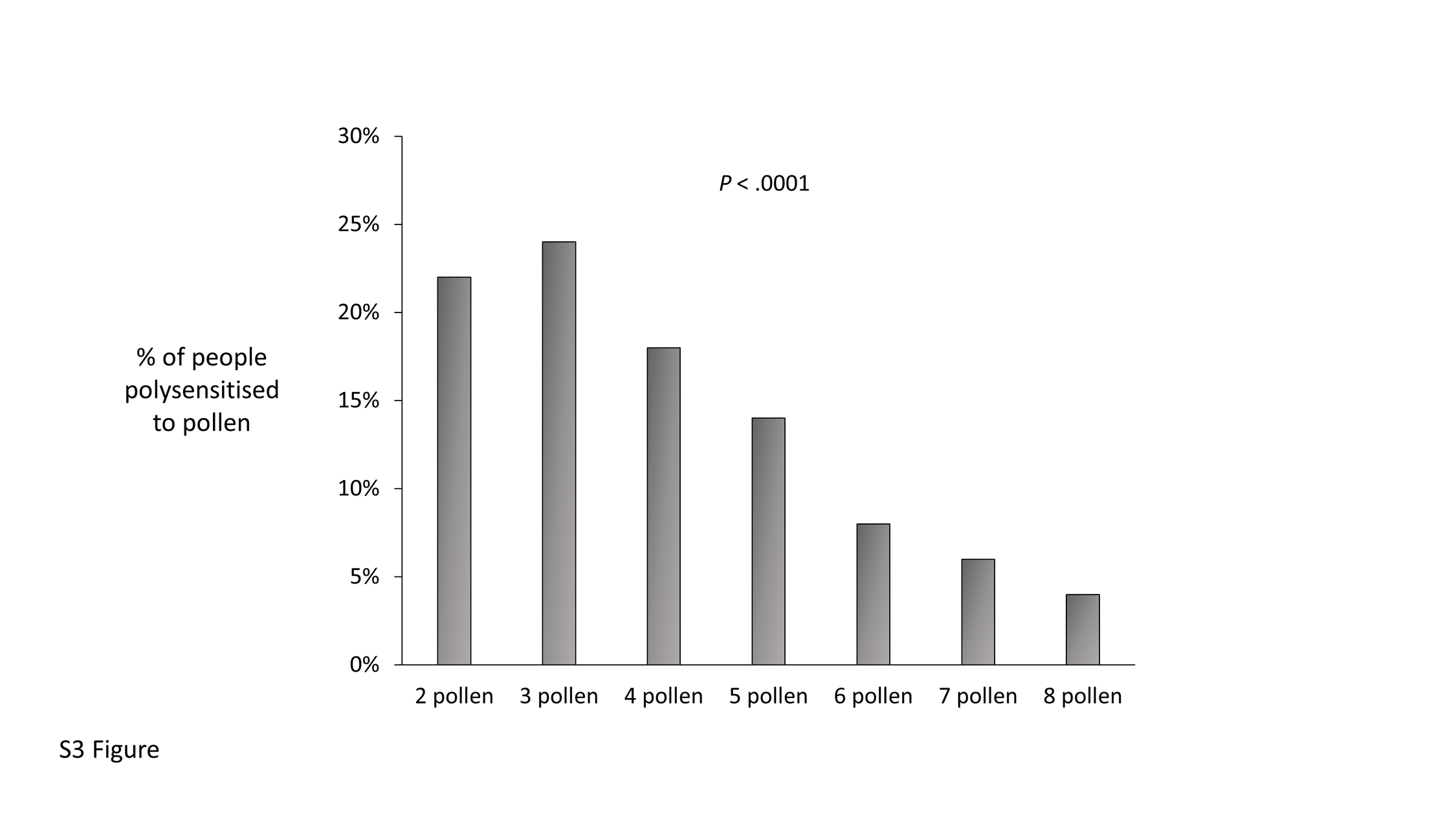

Supplement: S3 Fig — (TIF) [file pone.0255305.s003.tif]

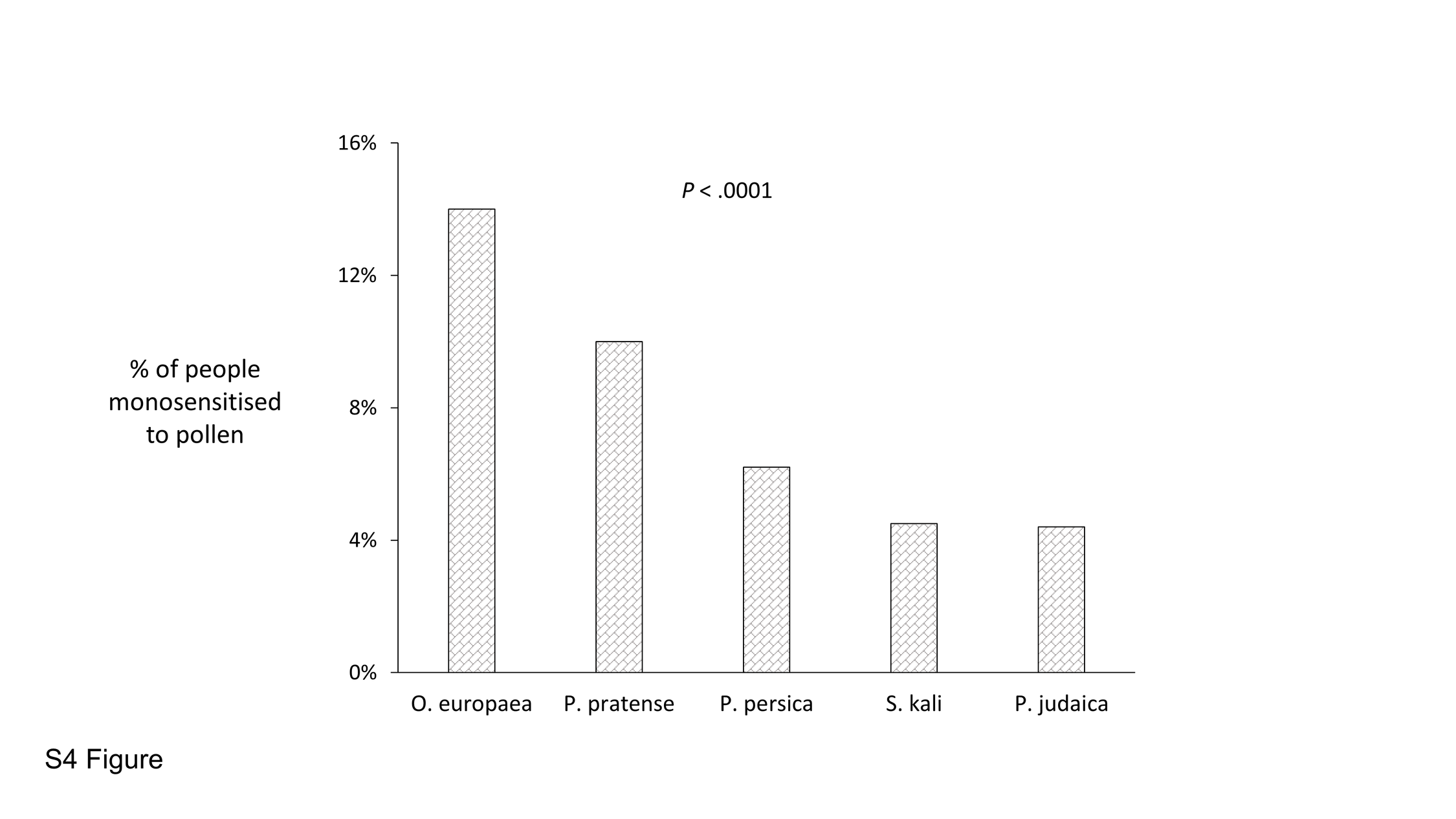

Supplement: S4 Fig — (TIF) [file pone.0255305.s004.tif]

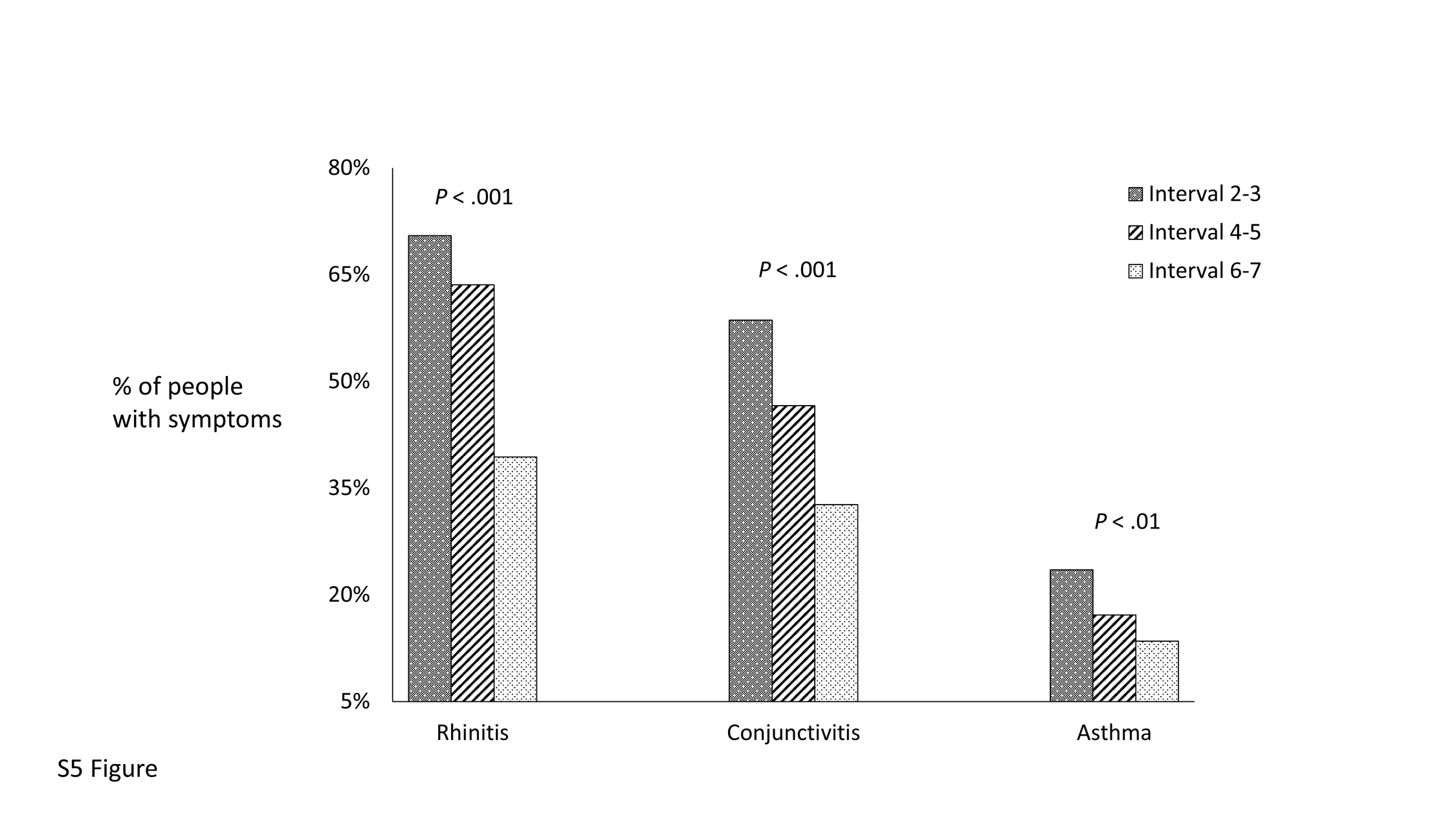

Supplement: S5 Fig — Interval 2–3: 21–40 y.o., interval 4–5: 41–60 y.o., interval 6–7: 61–83 y.o. (TIF) [file pone.0255305.s005.tif]
