# Supplementary material for: Subjects develop tolerance to Pru p 3 but respiratory allergy to Pru p 9: A large study group from a peach exposed population
Source: PLoS One. 2021 Aug 19;16(8):e0255305. doi: 10.1371/journal.pone.0255305 (PMC8376049; doi:10.1371/journal.pone.0255305)
Supplement: S1 Table — (DOCX) [file pone.0255305.s006.docx]

**S1 Table**: **demographic characteristics of the study group.**

| n = 716 |  |
| --- | --- |
| Males | 36% |
| Females | 64% |
| Agriculture | 80% |
| Services | 7% |
| Houseworker | 4% |
| Students | 5% |
| Fruit industry | 4% |
