# Supplementary material for: Subjects develop tolerance to Pru p 3 but respiratory allergy to Pru p 9: A large study group from a peach exposed population
Source: PLoS One. 2021 Aug 19;16(8):e0255305. doi: 10.1371/journal.pone.0255305 (PMC8376049; doi:10.1371/journal.pone.0255305)
Supplement: S2 Table — M: male, F: female. (DOCX) [file pone.0255305.s007.docx]

**S2 Table**. **Clinical characteristics of cases with OAS**

| **Case** | **Age** | **Gender** | **Clinical characteristics** | **Culprit food(s)** |
| --- | --- | --- | --- | --- |
| 1 | 37 | M | Oral pruritus | Banana |
| 2 | 43 | M | Oral pruritus, abdominal pain | Melon |
| 3 | 60 | M | Tongue pruritus, labial angioedema | Melon |
| 4 | 45 | M | Tongue angioedema | Kiwi, melon, pineapple |
| 5 | 29 | M | Tongue pruritus, labial angioedema | Melon |
| 6 | 23 | M | Tongue angioedema, sneezing, rhinorrhoea | Kiwi |
| 7 | 26 | M | Tongue pruritus, labial angioedema | Tomato, peanut, sunflower seed |
| 8 | 34 | M | Labial angioedema, oral pruritus | Tomato |
| 9 | 43 | M | Tongue pruritus, labial angioedema | Pineapple, melon |
| 10 | 49 | M | Tongue angioedema, difficulty swallowing | Walnut |
| 11 | 29 | F | Oral pruritus | Pineapple |
| 12 | 51 | M | Tongue pruritus, labial angioedema | Pineapple, melon |
| 13 | 51 | M | Oral pruritus | Kiwi |
| 14 | 36 | F | Oral pruritus, tongue angioedema | Walnut |
| 15 | 38 | M | Oral pruritus | Banana |
| 16 | 59 | M | Tongue angioedema, difficulty swallowing | Pineapple |
| 17 | 40 | M | Oral pruritus, abdominal pain | Peanut |
| 18 | 43 | M | Tongue pruritus, labial angioedema | Pineapple, melon, peanut |
| 19 | 45 | F | Oral pruritus, tongue angioedema | Kiwi, banana |
| 20 | 44 | M | Oral pruritus, tongue angioedema | Pineapple, melon |
| 21 | 39 | M | Oral pruritus | Kiwi, melon, watermelon |
| 22 | 50 | F | Pruritus, tongue angioedema | Melon |
| 23 | 47 | F | Oral pruritus, tongue angioedema | Pecan |
| 24 | 42 | M | Tongue pruritus, labial angioedema | Pineapple, melon |
| 25 | 38 | M | Oral and nasal pruritus | Kiwi, walnut |
| 26 | 68 | M | Oral pruritus | Melon |
| 27 | 44 | M | Oral pruritus, tongue angioedema | Pineapple, kiwi |
| 28 | 25 | M | Tongue pruritus, labial angioedema | Peanut, kiwi |
| 29 | 42 | M | Oral pruritus | Pineapple |
| 30 | 36 | F | Tongue pruritus, labial angioedema | Kiwi |
| 31 | 47 | M | Oral pruritus | Kiwi |
| 32 | 43 | M | Oral pruritus, tongue angioedema | Melon, fig |
| 33 | 33 | M | Oral pruritus | Melon |
| 34 | 48 | F | Oral pruritus | Pineapple |
| 35 | 39 | F | Oral pruritus, abdominal pain | Peanut, walnut |
| 36 | 42 | M | Oral pruritus | Melon |
| 37 | 66 | M | Oral pruritus | Melon |
| 38 | 40 | M | Oral pruritus, tongue angioedema | Melon, date |
| 39 | 46 | M | Oral and nasal pruritus | Pineapple, melon |
| 40 | 47 | M | Tongue pruritus, labial angioedema | Pineapple, kiwi, walnut |
| 41 | 56 | M | Tongue pruritus, labial angioedema | Pineapple, melon |
| 42 | 38 | M | Oral pruritus | Melon |
| 43 | 67 | M | Pharyngeal pruritus, abdominal pain | Melon |
| 44 | 30 | M | Oral and pharyngeal pruritus | Melon |
| 45 | 57 | M | Labial angioedema | Pineapple |
| 46 | 36 | M | Oral pruritus | Melon |
| 47 | 45 | M | Oral pruritus, tongue angioedema | Melon |
| 48 | 27 | M | Pharyngeal pruritus | Melon |
| 49 | 48 | M | Oral pruritus | Walnut, date, sunflower seed |
| 50 | 22 | F | Oral pruritus, labial angioedema | Melon, almond, tomato |
| 51 | 45 | M | Oral pruritus, tongue angioedema | Kiwi |
| 52 | 29 | M | Oral pruritus, tongue angioedema | Melon |
| 53 | 47 | M | Oral pruritus, labial angioedema | Melon, kiwi |
| 54 | 38 | M | Tongue pruritus, labial angioedema | Peanut, walnut |
| 55 | 68 | M | Oral pruritus, tongue angioedema | Peanut |
| 56 | 28 | M | Oral pruritus | Pineapple |
| 57 | 32 | M | Oral pruritus, labial angioedema | Peanut |
| 58 | 50 | F | Oral pruritus, tongue angioedema | Melon, banana |
| 59 | 42 | F | Pharyngeal pruritus, abdominal pain | Apple, peanut |
| 60 | 33 | M | Oral pruritus, tongue angioedema | Walnut, aubergine |
| 61 | 39 | M | Oral pruritus, labial angioedema | Melon, pineapple |
| 62 | 21 | F | Tongue pruritus | Kiwi |
| 63 | 38 | M | Oral pruritus, cough, wheezing | Sunflower seed |
| 64 | 34 | M | Pharyngeal pruritus | Walnut |
| 65 | 58 | F | Tongue pruritus, wheezing | Melon |
| 66 | 51 | M | Oral pruritus, labial angioedema | Peanut, pineapple, kiwi |
| 67 | 30 | M | Labial angioedema, tongue pruritus | Melon |
| 68 | 27 | M | Oral pruritus, labial angioedema | Walnut |
| 69 | 47 | M | Oral pruritus | Banana |
| 70 | 63 | M | Oral pruritus, labial angioedema | Melon, artichoke |
| 71 | 42 | M | Tongue pruritus, labial angioedema | Melon |
| 72 | 26 | F | Oral pruritus, abdominal pain | Peanut, apple |
| 73 | 22 | F | Pruritus, labial angioedema | Melon |
| 74 | 38 | M | Oral pruritus | Melon |
| 75 | 45 | F | Tongue pruritus | Walnut |
| 76 | 42 | M | Oral pruritus | Melon |
| 77 | 24 | M | Labial angioedema, tongue pruritus | Pineapple, sunflower seed |
| 78 | 43 | M | Oral pruritus, labial angioedema | Melon, banana |
| 79 | 61 | M | Oral pruritus | Melon, walnut |
| 80 | 47 | M | Labial angioedema, tongue pruritus | Walnut |
| 81 | 37 | F | Labial angioedema, tongue pruritus | Melon, watermelon, walnut |
| 82 | 26 | M | Oral pruritus, labial angioedema | Melon, banana |
| 83 | 35 | M | Oral pruritus | Peanut, melon, watermelon, kiwi |
| 84 | 40 | M | Labial angioedema, tongue pruritus | Melon |
| 85 | 83 | M | Oral pruritus, labial angioedema | Peanut, walnut |
| 86 | 56 | M | Labial angioedema, tongue pruritus | Melon, pineapple |
| 87 | 55 | M | Oral pruritus, labial angioedema | Melon |
| 88 | 25 | M | Labial angioedema, tongue pruritus | Peanut, almond, walnut |

M: male, F: female
