# Supplementary material for: Subjects develop tolerance to Pru p 3 but respiratory allergy to Pru p 9: A large study group from a peach exposed population
Source: PLoS One. 2021 Aug 19;16(8):e0255305. doi: 10.1371/journal.pone.0255305 (PMC8376049; doi:10.1371/journal.pone.0255305)
Supplement: S3 Table — (DOCX) [file pone.0255305.s008.docx]

|  |  |  | |  | |  | |  |
| --- | --- | --- | --- | --- | --- | --- | --- | --- |
|  |  | Symptomatic  to peach fruit | | Tolerant  to peach fruit | | *P* value | |  |
| Age  (years± SD) | 36±12 | | 37.5 ±13 | | .89 | |  |  |
| Pru p 3 IgE  (kU/L) | 6±2 | | 5±2 | | .84 | |  |  |
| Female | 71% | | 59% | | .45 | |  |  |
| *P. pratense* | 53% | | 59% | | .70 | |  |  |
| *O.europaea* | 65% | | 77% | | .38 | |  |  |
| *P. acerifolia* | 35% | | 18% | | .22 | |  |  |
| *C. arizonica* | 25.30% | | 36% | | .38 | |  |  |
| *A. vulgaris* | 35% | | 32% | | .81 | |  |  |
| *P. judaica* | 29% | | 41% | | .45 | |  |  |
| *P. persica pollen* | 65% | | 91% | | **.04** | |  |  |
| *Pru p 9* | 6% | | 41% | | **.01** | |  |  |
| *S. kali* | 18% | | 45% | | .06 | |  |  |
| *B. berrucosa* | 35% | | 23% | | .38 | |  |  |
| Apple | 35% | | 14% | | .11 | |  |  |
| Banana | 6% | | 3% | | .24 | |  |  |
| Peanut | 35% | | 27% | | .59 | |  |  |
| Almond | 6% | | 4.50% | | .85 | |  |  |
| Walnut | 18% | | 14% | | .73 | |  |  |
| Sunflower seed | 6% | | 14% | | .42 | |  |  |
| Tomato | 29% | | 18% | | .40 | |  |  |
| Peach fruit | 76.50% | | 82% | | .68 | |  |  |
| Kiwi | 0% | | 14% | | .11 | |  |  |
| Melon | 29% | | 23% | | .63 | |  |  |
| Mustard | 0% | | 9% | | .20 | |  |  |
| Pineapple | 0% | | 0% | |  | |  |  |

**S3 Table**: **Characteristics and sensitisation to pollen and food of subjects with SIgE to Pru p 3.**
