# Supplementary material for: Subjects develop tolerance to Pru p 3 but respiratory allergy to Pru p 9: A large study group from a peach exposed population
Source: PLoS One. 2021 Aug 19;16(8):e0255305. doi: 10.1371/journal.pone.0255305 (PMC8376049; doi:10.1371/journal.pone.0255305)
Supplement: S4 Table — F: female, M: male, OAS: oral allergy syndrome. (DOCX) [file pone.0255305.s009.docx]

**S4 Table. Characteristics of cases tolerant to peach fruit with positive SIgE to Pru p 3.**

| **Case** | **Age** | **Gender** | **IgE**  **Pru p 3**  **kU_A_/L** | **Intake of other fruits and nuts** |
| --- | --- | --- | --- | --- |
| 1 | 40 | F | 0.4 | Tolerance to Peach without skin, apple, peanut, walnut, cashew and watermelon |
| 2 | 32 | M | 2 | Tolerance to Peach without skin, plum, banana, peanut, almond and walnut |
| 3 | 23 | M | 3 | Tolerance to Peach with skin, peanut, walnut, melon and watermelon |
| 4 | 31 | M | 4.12 | Tolerance to Juice peach, banana and almond |
| 5 | 32 | F | 0.36 | Tolerance to Peach with skin, OAS with peanut, walnut and sunflower seed |
| 6 | 50 | M | 0.75 | Tolerance to Peach without skin, OAS with banana, and melon |
| 7 | 25 | F | 1.98 | Tolerance to Peach, pistachio and tomato. OAS with melon |
| 8 | 27 | F | 10.4 | Tolerance to Peach, banana, kiwi, peanut and cashew. OAS with walnut |
| 9 | 45 | M | 1.5 | Tolerance to Peach without skin, apple with skin, strawberry and orange. OAS with melon |
| 10 | 44 | F | 6 | Tolerance to Peach without skin. OAS with apple, melon, banana and pineapple |
| 11 | 34 | F | 2.3 | Tolerance to Peach with skin, peanut and walnut |
| 12 | 50 | F | 0.47 | Tolerance to Peach with skin, banana, orange, peanut |

F: female, M: male, OAS: oral allergy syndrome.
