# Supplementary material for: Subjects develop tolerance to Pru p 3 but respiratory allergy to Pru p 9: A large study group from a peach exposed population
Source: PLoS One. 2021 Aug 19;16(8):e0255305. doi: 10.1371/journal.pone.0255305 (PMC8376049; doi:10.1371/journal.pone.0255305)
Supplement: S5 Table — CI: confidence interval; OR: odds ratio; SPT: skin prick test. (DOCX) [file pone.0255305.s010.docx]

**S5 Table.** **Multivariable logistic regression analysis for peach-induced food allergy or respiratory allergy**

| **Variables associated with outcomes 1 and 2** | **OR** | **95% CI** |
| --- | --- | --- |
| **1. Peach-induced food allergy** |  |  |
| Female gender | 2.78 | 1.01–7.66 |
| SPT-positive to apple | 2.75 | 0.77–9.84 |
| Positive IgE to Pru p 3 | 12.00 | 4.47–32.22 |
| SPT-positive to any food | 3.74 | 1.48–9.47 |
| **2. Peach tree-induced respiratory allergy** | | |
| Aged 21–40 years (reference age 61–83 years) | 2.02 | 1.20–3.42 |
| Aged 41–60 years (reference age 61–83 years) | 2.36 | 1.42–3.91 |
| Female gender | 1.95 | 1.33–2.86 |
| SPT-positive to *Olea europaea* | 2.68 | 1.47–487 |
| SPT-positive to any pollen | 4.17 | 0.99–6.78 |

CI: confidence interval; OR: odds ratio; SPT: skin prick test
