# Supplementary material for: Subjects develop tolerance to Pru p 3 but respiratory allergy to Pru p 9: A large study group from a peach exposed population
Source: PLoS One. 2021 Aug 19;16(8):e0255305. doi: 10.1371/journal.pone.0255305 (PMC8376049; doi:10.1371/journal.pone.0255305)
Supplement: S6 Table — R: rhinitis, RC: rhinoconjuctivitis, A: Asthma, Pollens for skin prick testing: O: Olea europaea, C: Cupresus arizonica, Pj: Parietaria judaica, Pp: Phleum pratense, S: Salsola kali, Pa: Platanus acerifolia, Av: Artemisia vulgaris. PTP: peach tree pollen, SPT: skin prick test, NPT: nasal provocation test. +: positive. -: negative. Positive NPT: >20% nasal volume fall and compatible symptoms. (DOCX) [file pone.0255305.s011.docx]

**S6 Table**. **Characteristics of cases that underwent nasal provocation test to peach tree pollen and Pru p 9**

| Case | Age | Gender | Clinical characteristics | SPT +  pollen | SPT  PTP | SPT  Pru p 9 | NPT  PTP | NPT  Pru p 9 |
| --- | --- | --- | --- | --- | --- | --- | --- | --- |
| 1 | 26 | F | RC | O, C, Pj | + | + | + | - |
| 2 | 50 | M | RC | Pp, S, Pj | + | + | + | + |
| 3 | 43 | F | RC+A | O, Pp | + | + | + | + |
| 4 | 57 | F | RC+A | O, Pp, Pj, S, Pa | + | + | + | + |
| 5 | 58 | F | R+A | O, S, Pj | + | + | + | + |
| 6 | 29 | M | RC | O, Pp | + | + | - | + |
| 7 | 32 | M | RC | Pp, S, Pj | + | + | + | + |
| 8 | 47 | M | RC | O, Pj | + | + | + | + |
| 9 | 53 | F | RC+A | O, S | + | + | + | - |
| 10 | 29 | F | RC | O, Pp, C, Pj | + | + | + | + |
| 11 | 58 | F | RC | Pp, S, Av | + | + | + | + |
| 12 | 38 | M | R+A | O, Pp, Pa | + | + | - | + |
| 13 | 53 | M | RC | Pp, S, Av | + | + | + | + |
| 14 | 21 | F | R | O, Pp, Pa, Av | + | + | + | + |
| 15 | 35 | F | RC | O, S | + | + | + | + |
| 16 | 40 | M | RC | O, Pa | + | + | + | + |
| 17 | 49 | F | RC+A | O, Pp, Pj | + | + | + | + |
| 18 | 24 | M | RC | Pp, S, Pj | + | + | + | + |
| 19 | 46 | F | RC | O, Pp, C, Av | + | + | + | + |
| 20 | 38 | F | RC | O, Pa, C | + | + | + | + |

R: rhinitis, RC: rhinoconjuctivitis, A: Asthma, Pollens for skin prick testing: O: *Olea europaea*, C: *Cupresus arizonica*, Pj: *Parietaria judaica*, Pp: *Phleum pratense*, S: *Salsola kali*, Pa: *Platanus acerifolia*, Av: *Artemisia vulgaris*.

PTP: peach tree pollen, SPT: skin prick test, NPT: nasal provocation test

+: positive. - : negative.

Positive NPT: >20% nasal volume fall and compatible symptoms.
