# Supplementary material for: Subjects develop tolerance to Pru p 3 but respiratory allergy to Pru p 9: A large study group from a peach exposed population
Source: PLoS One. 2021 Aug 19;16(8):e0255305. doi: 10.1371/journal.pone.0255305 (PMC8376049; doi:10.1371/journal.pone.0255305)
Supplement: S1 Appendix — (DOCX) [file pone.0255305.s012.docx]

**S1 Appendix**

**Quantitative variables**: age in years, specific IgE antibodies to Pru p 3, N months when symptoms appeared, number of pollens yielding positive skin prick tests (1 to 8), N food allergens to which participants developed a positive response (1 to 12), years living in the region.

**Qualitative variables**: age groups (21–40 years, 41-60 years, 61-83 years); months (January to December); gender (male/female); place of birth (in the region of study/in another region), skin prick test results (positive/negative); asthma (yes/no); rhinitis (yes/no); asthma + rhinitis (yes/no); conjunctivitis (yes/no); peach intake (yes/no); if yes, the quantity per year (< 5 kg, 5 to <10 kg, 10 to <15 kg, 15 kg to <20 kg, > 20 kg). For food allergy the following clinical entities were considered: oral allergy syndrome (OAS) to any fruit with good tolerance to peach (yes/no), OAS to any fruit including peach (yes/no), urticaria to any fruit with good tolerance to peach (yes/no), urticaria with peach (yes/no), anaphylaxis to any fruit with good tolerance to peach (yes/no), anaphylaxis with peach (yes/no), occupation.
