# Supplementary material for: Subjects develop tolerance to Pru p 3 but respiratory allergy to Pru p 9: A large study group from a peach exposed population
Source: PLoS One. 2021 Aug 19;16(8):e0255305. doi: 10.1371/journal.pone.0255305 (PMC8376049; doi:10.1371/journal.pone.0255305)
Supplement: S2 Appendix — (DOCX) [file pone.0255305.s013.docx]

**S2 Appendix**

**Allergens tested.**

-Pollen allergens: *P. pratense, O. europaea, P. acerifolia, C. arizonica, A. vulgaris, P. judaica, P. persica, Pru p 9, S. kali* and *B. berrucosa.*

- Perennial allergens: *D. pteronyssinus, A. alternata*, cat and dog dander.

- Food allergens: Apple, Banana, Peanut, Almond, Walnut, Sunflower seed, Tomato, Peach fruit, Kiwi, Melon, Mustard and Pineapple.
